# Tricellular Tight Junctions in the Inner Ear

**DOI:** 10.1155/2016/6137541

**Published:** 2016-04-18

**Authors:** Shin-ichiro Kitajiri, Tatsuya Katsuno

**Affiliations:** Department of Otolaryngology-Head and Neck Surgery, Graduate School of Medicine, Kyoto University, Kyoto 606-8507, Japan

## Abstract

Tight junctions (TJs) are structures that seal the space between the epithelial cell sheets. In the inner ear, the barrier function of TJs is indispensable for the separation of the endolymphatic and perilymphatic spaces, which is essential for the generation and maintenance of the endocochlear potential (EP). TJs are formed by the intercellular binding of membrane proteins, known as claudins, and mutations in these proteins cause deafness in humans and mice. Within the epithelial cell sheet, however, a bound structure is present at the site where the corners of three cells meet (tricellular tight junctions (tTJs)), and the maintenance of the barrier function at this location cannot be explained by the claudins alone. Tricellulin and the angulin family of proteins (angulin-1/LSR, angulin-2/ILDR1, and angulin-3/ILDR2) have been identified as tTJ-associated proteins. Tricellulin and ILDR1 are localized at the tTJ and alterations in these proteins have been reported to be involved in deafness. In this review, we will present the current state of knowledge for tTJs.

## 1. Introduction

Sound vibrations are converted into nerve action potentials in the inner ear [1]. For this process to occur, the strict compartmentalization of the cochlea is necessary. The structure of the inner ear can be broadly separated into two compartments: the endolymphatic space (endolymph) and perilymphatic space (perilymph). Within the cochlea, the endolymph and perilymph have an entirely different composition. The endolymph has a higher electrical potential (endocochlear potential (EP)) and a higher potassium concentration, when compared to the perilymph [2]. The barrier function of the epithelial cell sheet prevents paracellular permeability, and the separation of these two compartments is essential for the maintenance of their differences in composition [3]. Tight junctions (TJs) are intercellular junctions that play a major role in epithelial barrier function. TJs are formed by the TJ strand, which is a fibril-like structure consisting of tight junction-associated proteins from both of the adjacent plasma membranes [4–9]. The claudins are integral membrane proteins, identified as response molecule of barrier function, which form the TJ that seals the space between neighboring epithelial cells. To date, 24 claudins have been identified [10–12] and at least 10 of these are expressed in the inner ear [13]. Claudin-1, claudin-2, claudin-3, claudin-9, claudin-10, claudin-12, claudin-14, and claudin-18 are expressed in the organ of Corti. In addition, claudin-8 is expressed in Rissner's membrane, the spiral limbus, and the marginal cells of the stria vascularis and only claudin-11 is expressed in the basal cells of the stria vascularis [13]. The combination of these claudins is thought to be important for barrier function in the inner ear, and three of these claudins (claudin-9, claudin-11, and claudin-14) are reported to be critical for hearing [14–20]. Mutations in these proteins cause deafness in humans and mice. Claudin-11 (cldn-11) knockout mice demonstrate hearing loss as a result of reduced EP [14, 15]. Interestingly, mutations in human* CLDN14* cause profound, congenital deafness DFNB29 [16]. Both cldn-9 mutant mice and cldn-14 null mice are deaf, due to the rapid degeneration of cochlear hair cells shortly after birth, but do not display reduced EP [17, 18]. These phenotypes are considered to be associated with local disturbances in ionic balance within the inner ear. Therefore, the epithelial barrier that is formed by TJs is significantly involved in inner ear function.

## 2. The Tricellular Tight Junction (tTJ) in the Inner Ear

**Figure 1:**
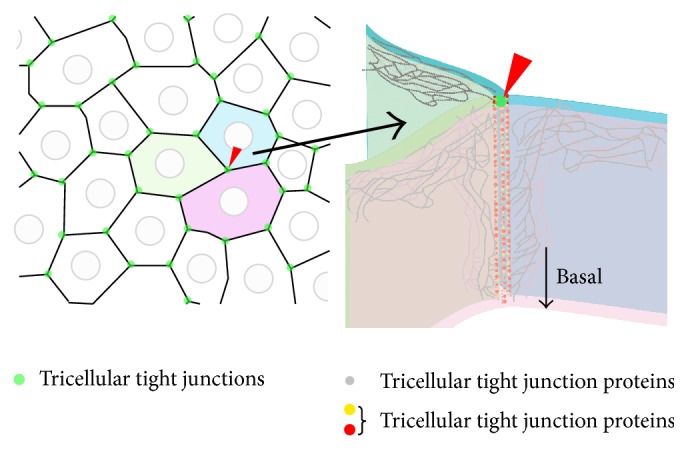
Schematic image of the tricellular tight junction (tTJ). The TJs between two cells (bicellular TJ, bTJ) function to seal off the intercellular space (black lines). However, the corner where three cells meet (green dots) cannot be occluded by bTJ proteins alone and a central “tube” may exist. tTJ proteins (red dots and yellow dots) are localized at this site and play a role in providing a barrier.

**Figure 2:**
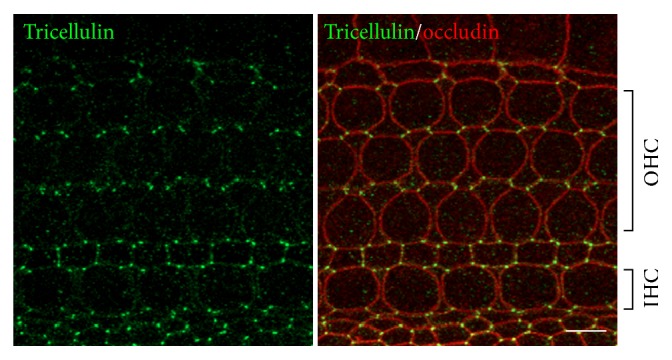
Localization of tricellulin and occludin in the organ of Corti. Double immunofluorescence microscopy of the organ of Corti of postnatal day 3 C57BL/6 mice using anti-tricellulin pAb (green) and anti-occludin mAb (red). Tricellulin is localized at tricellular contacts where three cells meet. Occludin, a tight junction-associated membrane protein, distributed to cell-cell contacts not only at tTJ but also at bicellular TJ. OHC: outer hair cell; IHC: inner hair cell. Bars, 5 *μ*m.

The epithelial cell sheet contains TJs between cells and functions as a barrier [19, 20]. However, at the apex of polygon-shaped cells, the corners of three epithelial cells abut against each other and this results in a structure that differs from normal TJs that are formed between two cells (Figure 1). At the apex of the three cells, the most apical strands of the horizontal TJs extend to the center of the corner, then turn, and grow in the basal direction. They are nearly attached to each other to form vertical strands. As a result, a narrow and long tube-like structure is formed in the extracellular space at the corner. This tubular structure is called a tTJ and is thought to reduce the free diffusion of solutes to ensure a sufficient paracellular barrier [21]. tTJs were first reported in the 1970s as structures visible in electron micrographs. However, the molecular entities comprising tTJs were unclear for a long time. The first molecule found to play a role in tTJs was tricellulin [22], and three more proteins, LSR/angulin-1, ILDR1/angulin-2, and ILDR2/angulin-3, have recently been identified [23, 24]. Tricellulin, LSR, and ILDR1 are each expressed in the inner ear and both tricellulin and ILDR1 are localized at the tTJ in the organ of Corti (Figure 2). These two genes are responsible for human deafness DFNB49 and DFNB42, respectively [25, 26].

## 3. Tricellulin and the tTJ

Tricellulin was first identified in a genetic screen for factors that are suppressed during the forced expression of the snail transcription factor, which is involved in the epithelial to mesenchymal transition in cultured mouse epithelial cells. Tricellulin is a four-pass transmembrane protein belonging to the MARVEL family, which also includes occludin (ocln), a TJ membrane protein that was identified before the role of the claudin proteins was determined [22]. Tricellulin is localized at the tTJ of epithelial cells in several locations, including those of the inner ear (Figure 2). Intercellular barrier function was found to be diminished following the RNAi knockdown of tricellulin expression in cultured epithelial cells, indicating that tricellulin is essential for sufficient barrier function [22]. Tricellulin also displays similar properties to the claudin proteins in providing a barrier against electrical conductance and permeability for ionic and uncharged solutes [22, 27, 28]. Mutations in the gene encoding tricellulin are also causative factors of human hereditary deafness DFNB49 [25]. Mice with a knockin of this human deafness mutation exhibit severe hearing loss caused by the degeneration of hair cells, similar to the phenotype observed with claudin-9 and claudin-14 deficiency [17, 19]. Furthermore, tricellulin was found to no longer be localized at the tTJ in the mutant mice. The morphology of the TJ can be visualized as a strand by the freeze-fracture replica method. In tTJs, the TJ strands extend deep into the basal side; however, in the organ of Corti of these mutant mice, the TJ strands remain separated from each other and the short TJ strands packed orthogonally are sparse. Therefore, tricellulin is thought to be necessary for the maintenance of the tTJ structure in the inner ear [29].

## 4. Occludin (Ocln)

Occludin is a member of the same protein family as tricellulin; however, it is localized to both tTJs and normal TJs [30, 31]. Occludin has been reported to affect the localization of tricellulin [32]. Occludin knockout mice exhibit a similar phenotype to tricellulin knockin mice, with apoptosis of the hair cells and degeneration of the organ of Corti. In these knockout mice, tricellulin is no longer localized to the tTJ, which is believed to be the cause of the observed deafness. This demonstrates that an interplay between the MARVEL family members is necessary for determining the localization of tricellulin and that the concentration of tricellulin at the tTJ is important for its function [33].

## 5. ILDR1 and the Angulin Family

Following the identification of tricellulin, a membrane protein known as lipolysis-stimulated lipoprotein receptor (LSR) was discovered. LSR was found through the expression cloning of an epithelium-derived cDNA library using localization to intercellular junctions as a marker and identified as a component of the tTJ [23]. RNAi knockdown of LSR expression in cultured epithelial cells was shown to decrease transepithelial electrical resistance (TER), suggesting that LSR is involved in barrier function. Interestingly, in LSR knockdown cells, tricellulin is dispersed across the cell membrane, rather than being concentrated at the tTJ, indicating that LSR regulates the localization of tricellulin to the tTJ [23]. LSR has two homologous genes known as immunoglobulin-like domain-containing receptor 1 and immunoglobulin-like domain-containing receptor 2 (ILDR1 and ILDR2). Together with LSR, these three proteins regulate the localization of tricellulin at the tTJ. These proteins form the angulin family, with LSR, ILDR1, and ILDR2 also known as angulin-1, angulin-2, and angulin-3, respectively [24]. Of these three angulin proteins, mutations in ILDR1 (angulin-2) have been identified as causes of human hereditary deafness [26]. By immunostaining, only ILDR1 was found to be expressed in the organ of Corti [24]. Because angulins can recruit tricellulin to the tTJ, the mutations in angulin-2/ILDR1 may cause hearing loss via changes in tricellulin localization. Surprisingly, however, tricellulin was found to localize at the tTJ in ILDR1 knockout mice, although its distribution along the depth of the tricellular contacts was subtly affected [34, 35]. Angulin-1/LSR is not normally expressed in the organ of Corti; however, its expression is seen when angulin-2/ILDR1 is deficient, and tricellulin is thought to be recruited to the tTJ as a result. This clearly demonstrates that compensatory functions exist between the angulin family members. However, the organ of Corti degenerated even when tricellulin was localized at the tTJ by angulin-1/LSR, indicating that there are also functional differences between the angulin family members [35].

## 6. EP Generation and Hair Cell Degeneration

**Figure 3:**
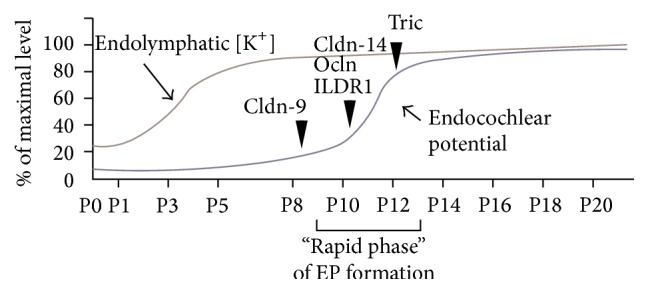
Time course of EP and endolymphatic [K^+^] elevation (modified from [38–41]) and hair cell degeneration in mice with mutations in TJ proteins. The arrowheads indicate the starting time for hair cell degeneration in each of the mutant mice models: cldn-9: claudin-9 mutant mice [18]; cldn-14: claudin-14 knockout mice [17]; ocln: occludin deficient mice [33]; ILDR1: ILDR1 null mice [34, 35]; Tric: tricellulin knockin mice [29] and knockout mice [36].

Mutant mice have been shown to share deafness phenotypes with unchanged EP and hair cell degeneration all occurring within the same timeframe [17, 18, 29, 34, 36]. EP is likely to be related with hair cell degeneration in these mice. First, in explant culture condition, in which EP does not exist, the hair cell degeneration was not observed. Second, in double mutant mice with pou3f4-deficient mice, in which EP does not generate, hair cell degeneration was reduced [17, 18]. These results indicate that hair cell degeneration is triggered by changes to extracellular conditions. There may, therefore, be a common mechanism underlying deafness in these models, which is associated with local disturbances in ionic balance within the inner ear caused by leakage of K^+^ and Na^+^ ions or small molecules such as ATP [37]. In fact, it has reported that the concentration of K^+^ ions in perilymph is slightly, but significantly, increased in cldn-9 mutant mice [18]. It might be involved in the viability of hair cells. In addition, the initiation of hair cell degeneration overlaps with the “rapid phase” of EP formation (Figure 3) [37–40]. There is difference in the timing of initiation of hair cell degeneration within “rapid phase” in mutant mice during EP formation. Furthermore, TJs not only play a role in providing a barrier function but also function as a selective barrier similar to a channel [6, 7, 22, 41–44]. This difference in timing for the initiation of hair cell degeneration might be caused by differences in paracellular permeability properties (the nature and amount of the molecules permitted through the barrier) associated with each of the TJ proteins.

## 7. Conclusion

Hearing loss due to the failure of the normal bicellular TJ has been extensively investigated in both humans and mice. In addition, the new field of tTJ study has shown that this structure is also deeply involved in inner ear function. The phenotypes of mice with mutations in tTJ-associated proteins were found to be similar to those of mice with mutations in bTJ-associated proteins. At present, the organ of Corti is known to express two tTJ-associated proteins, namely, tricellulin and ILDR1. Both of these proteins have been associated with human deafness and mouse models with mutations in these proteins display hair cell degeneration. Further studies will be useful to evaluate the mechanism of this hair cell degeneration.
